# Embedding research in health policy and systems in the Americas

**DOI:** 10.26633/RPSP.2017.68

**Published:** 2017-04-28

**Authors:** Etienne V Langlois, Nhan T Tran, Abdul Ghaffar, Ludovic Reveiz, Francisco Becerra-Posada

**Affiliations:** 1 Alliance for Health Policy and Systems Research World Health Organization Geneva Switzerland Alliance for Health Policy and Systems Research, World Health Organization, Geneva, Switzerland. Send correspondence to Etienne V. Langlois:; 2 Pan American Health Organization Pan American Health Organization Washington, DC United States of America Pan American Health Organization, Washington, DC, United States of America.

As countries develop and improve universal health coverage schemes globally, there is increasing recognition that health policymaking and health system strengthening need to be informed by robust research evidence (1, 2). In Latin America and the Caribbean, emerging and complex health challenges require actionable and context-sensitive evidence to improve the responsiveness of health systems (3). This movement towards evidence-informed policymaking also calls for research that addresses key priorities identified by policymakers and stakeholders in the region.

To stimulate this type of research the World Health Organization (WHO) developed *Changing Mindsets*, a strategy on health policy and systems research that advocates for thoroughly embedding research into health system decision-making (4). Recognizing the importance of early and active engagement of policymakers, the *World Health Report 2013* (1) focused specifically on research for universal health coverage and called for more demand-driven research globally. Furthermore, there is a growing interest in the co-development of research and the engagement of policymakers in various empirical endeavors worldwide (5). Recent evidence suggests that partnerships between researchers and policymakers increase the relevance of scientific findings and improve the use of research to support health policy and practice (6, 7). Ongoing collaboration and engagement of policymakers also seem better suited to real-world policymaking and complex health system dynamics (8).

To promote this collaborative approach, the Alliance for Health Policy and Systems Research (the Alliance), an international partnership hosted by WHO, developed an innovative model of embedded research led by policymakers and health systems decision-makers in low- and middle-income countries (LMICs). The Alliance understands embedded research as a process driven by health system decision-makers to address context-specific factors relevant to key policy priorities. As such, policymakers, program managers, and implementers act as co-principal investigators and play a fundamental role in planning and conducting the embedded research projects alongside the researchers. Policymakers and health system decision-makers are the actors who are best positioned to ensure that findings are integrated in real-time to support the development and implementation of health policies and the performance of health systems. For instance, embedded research has the potential to increase the use of evidence to successfully implement and scale-up proven health programs and policies, thus moving towards population health impact.

Since 2014, the Alliance, in collaboration with the Pan American Health Organization (PAHO), has implemented an embedded research initiative to support health policies and programs in Latin America and the Caribbean. The initiative, entitled “Improving Program Implementation through Embedded Research (iPIER),” aims to support the development of and demand for problem-focused and action-oriented research. The Alliance contributes its embedded research experience and PAHO brings its expertise with supporting research in the Region of the Americas to advocate for this innovative way of developing and using science.

In Latin America and the Caribbean, the embedded research approach was taken in response to the recognized need for more health systems research in the area (9) and the high potential for improved health policy implementation and effectiveness. During 2014–2015, the Alliance funded seven embedded research projects, and PAHO subsequently supported an additional five proposals. These embedded research initiatives were implemented in nine countries: Argentina, Bolivia, Brazil, Colombia, Chile, Mexico, Panama, Peru, and Saint Lucia. The Institute for Clinical Effectiveness and Health Policy (Buenos Aires, Argentina) also provided technical assistance to the research teams.

This special series of the *Pan American Journal of Public Health* presents the rationale that underpins the embedded research initiative, and the findings of 10 of these projects, conducted within various health system settings in Latin America and the Caribbean. The supplement provides critical knowledge emanating from research prioritized by decision-makers, in addition to documenting the embedded research process and outcomes. Findings and reflections put forth in this series also contribute to bridging the knowledge gap on the engagement process between policymakers and researchers, particularly in LMIC settings. In addition, the learnings provide greater evidence on the pathways through which research is linked to priority health systems and services in the Americas.

While the embedded research model has been implemented as a pilot in the Americas—with a second phase launched in September 2016—our long-term vision is that funders and governments worldwide will recognize the value of embedded research and will duly take on the responsibility of supporting this approach. The impact of the embedded research approach will only be realized if those responsible for supporting the implementation and scale-up of health program are willing to provide resources and consider research to be an integral part of programmatic implementation.

iPIER has empowered policymakers, program managers, and other decision-makers in the Americas to engage in and take ownership of research by driving the development of research questions and by using findings to improve decision-making. By promoting more relevant and demand-driven research, we believe the embedded research approach has the potential to make significant improvements to implementation and scale-up of health interventions and to promote universal health coverage in the Americas.
